# Down regulation of Chk1 by p53 plays a role in synergistic induction of apoptosis by chemotherapeutics and inhibitors for Jak2 or BCR/ABL in hematopoietic cells

**DOI:** 10.18632/oncotarget.9844

**Published:** 2016-06-06

**Authors:** Yoshihiro Umezawa, Tetsuya Kurosu, Hiroki Akiyama, Nang Wu, Ayako Nogami, Toshikage Nagao, Osamu Miura

**Affiliations:** ^1^ Department of Hematology, Graduate School of Medical and Dental Sciences, and Graduate School of Biomedical Science, Institute of Biomaterials and Bioengineering, Tokyo Medical and Dental University, Tokyo, Japan

**Keywords:** Chk1, p53, BCR/ABL, Jak2, chemotherapeutics

## Abstract

DNA-damaging chemotherapeutic agents activate apoptotic pathways in cancer cells. However, they also activate checkpoint mechanisms mainly involving Chk1 and p53 to arrest cell cycle progression, thus abbreviating their cytotoxic effects. We previously found that aberrant tyrosine kinases involved in leukemogenesis, such as BCR/ABL and Jak2-V617F, as well as Jak2 activated by hematopoietic cytokines enhance Chk1-mediated G2/M arrest through the PI3K/Akt/GSK3 pathway to confer resistance to chemotherapeutic agents, which was prevented by inhibition of these kinases or the downstream PI3K/Akt pathway. However, the possible involvement of p53 in regulation of Chk1-mediated G2/M checkpoint has remained to be elucidated. We demonstrate here that a dominant negative mutant of p53, p53-DD, increases Chk1-mediated G2/M checkpoint activation induced by chemotherapeutics and protects it from down regulation by inhibition of Jak2, BCR/ABL, or the PI3K/Akt pathway in hematopoietic model cell lines 32D and BaF3 or their transformants by BCR/ABL. Consistent with this, the p53 activator nutlin-3 synergistically induced apoptosis with chemotherapeutics by inhibiting Chk1-mediated G2/M arrest in these cells, including cells transformed by the T315I mutant of BCR/ABL resistant to various kinase inhibitors in clinical use. Further studies suggest that p53 may inhibit the Chk1 pathway by its transcription-dependent function and through mechanisms involving the proteasomal system, but not the PI3K/Akt/GSK3 pathway. The present study may shed a new light on molecular mechanisms for the therapy resistance of p53-mutated hematological malignancies and would provide valuable information for the development of novel therapeutic strategies against these diseases with dismal prognosis.

## INTRODUCTION

Chemotherapeutic agents generally induce DNA damages to activate apoptotic pathways in cancer cells [1]. However, DNA damages also elicit checkpoint responses that delay or arrest the cell cycle to allow DNA repair, thus counteracting chemotherapeutic effects [2, 3]. DNA damages induce G1/S arrest to prevent replication of damaged DNA or G2/M arrest to prevent progression of cells with damaged chromosomes into mitosis, which leads to a catastrophic cell death. The G2/M arrest is mainly mediated by activation of the serine/threonine kinase Chk1, which is activated by phosphorylation on S317 and S345 by the DNA damage-activated ATR kinase in response to genotoxic stress and inhibits the Cdc25 phosphatases, thus increasing the level of inhibitory phosphorylation of Cdc2 on Tyr15 and Thr14 to arrest the G2/M transition [2]. The Chk1 activation is down regulated and terminated through dephosphorylation by PP2A and other phosphatases as well as through its degradation via the ubiquitination/proteasomal system (UPS) [2].

On the other hand, the G1/S checkpoint is mainly mediated through the tumor suppressor p53, which inactivates the Cdk2 kinase mainly through induction of the Cdk inhibitor p21 expression [4, 5]. p53 also plays important roles in induction of apoptosis in response to cellular stresses, including DNA damages, through its transcription-dependent and -independent functions. In the absence of stress, p53 is tightly controlled by Mdm2, which associates with p53 to induce its ubiquitination and degradation. In response to cellular stress, including DNA damages, the p53 level is elevated by post-translational mechanisms that interfere with its interaction with Mdm2. For instance, activated ATR and Chk1 induce p53 expression by phosphorylating S15 and S20 on p53 to prevent its association with Mdm2 [4, 5]. Thus, Mdm2 inhibitors, such as nutlin-3, have been developed to induce p53 expression in the absence of cellular stress or to enhance its expression synergistically with cellular stress [6, 7]. p53 is the most frequently inactivated protein in human malignancies with about 50% of all solid tumors showing mutations or deletion in the p53 gene. Although p53 is mutated in only about 10% of hematopoietic malignancies, it is associated with a very poor prognosis [8]. It has been reported that p53 is also involved in the regulation of G2/M checkpoint by inhibiting Cdc2 through various mechanisms [9]. However, the possible involvement of p53 in regulation of Chk1-mediated G2/M checkpoint has remained to be elucidated.

The Jak family tyrosine kinase Jak2 is activated by hematopoietic cytokine receptors, such as those for IL-3 and erythropoietin (Epo), and plays a crucial role in regulation of survival and proliferation of hematopoietic cells by activating various intracellular signaling pathways, including the Ras/MEK/Erk and PI3K/Akt pathways, and STAT5 [10]. Thus, aberrant activation of Jak2 by the V617F mutation enhances survival and proliferation of hematopoietic cells and plays a crucial role in pathogenesis the Philadelphia chromosome (Ph)-negative myeloproliferative neoplasms (MPN), such as polycythemia vera and essential thrombocythemia [11]. On the other hand, the Ph-positive MPN chronic myeloid leukemia (CML) is caused by the constitutively-activated fusion tyrosine kinase BCR/ABL generated by a reciprocal t(9;22) (q34;q11.2) chromosomal translocation causing Ph, which also plays a critical role in pathogenesis of 30–40% of acute lymphoblastic leukemia (ALL) [12]. BCR/ABL also confers survival and proliferation advantages on hematopoietic cells by activating various intracellular signaling pathways similarly with cytokine receptor-activated Jak2 or Jak2-V617F. Various tyrosine kinase inhibitors (TKIs) that block the catalytic activity of BCR/ABL, such as imatinib, nilotinib, and dasatinib, have been in clinical use and have demonstrated unprecedented efficacy for treatment for CML or Ph-positive ALL [13, 14]. However, resistance to these TKIs develops in significant portions of patients under treatment, especially in those with CML in advanced stages or with Ph+ ALL, mostly due to the emergence of mutations in the BCR/ABL kinase domain. These mutations include the clinically most important T315I mutation in the tyrosine kinase domain of BCR/ABL, which confers a complete resistant to not only the first generation inhibitor imatinib but also to the second generation inhibitors nilotinib and dasatinib [13, 14]. The Jak kinase inhibitors, such as ruxolitinib, have also been in clinical use for Ph-negative MPN with rather limited efficacies [15].

We have previously revealed that the aberrant tyrosine kinases, such as BCR/ABL and Jak2-V617F, as well as hematopoietic cytokines, such as IL-3 and Epo, enhance Chk1-mediated cell cycle checkpoint activation by chemotherapeutics through inhibition of GSK3 by activating the PI3K/Akt pathway, thus protecting hematopoietic cells from induction of apoptosis [16, 17]. Thus, apoptosis induced by chemotherapeutics was synergistically enhanced by inhibition of these aberrant kinases or the PI3K/Akt pathway by clinically relevant inhibitors, such as imatinib or pictilisib (GDC-0941), through down regulation of Chk1-mediated cell cycle arrest [17]. We have also found that imatinib synergistically induced apoptosis of BCR/ABL-expressing cells caused with nutlin-3, which induces p53 expression by inhibiting its Mdm2-mediated degradation [18]. However, a possible role p53 may play in induction of apoptosis synergistically by the combined treatment with TKIs and chemotherapeutics in hematopoietic cells has remained to be evaluated.

In the present study, we demonstrate that a dominant negative mutant of p53, p53-DD, enhances Chk1-mediated G2/M checkpoint activation induced by chemotherapeutics and prevents its down regulation by inhibitors for Jak2, BCR/ABL, or the PI3K/Akt pathway in hematopoietic model cell lines 32D and BaF3 or their transformants by BCR/ABL. In accordance with this, the p53 inducer nutlin-3 down regulated Chk1-mediated G2/M checkpoint activation and induced apoptosis synergistically with chemotherapeutics in hematopoietic cells, including those transformed by the T315I mutant of BCR/ABL resistant to various TKIs. Further studies suggested that the proteasomal pathway, but not GSK3, may be involved in the down regulation of Chk1 activation by p53 through its transcription activating activity. Together these data indicate that p53 may enhance the cytotoxic effects of chemotherapeutics at least partly through down regulation of the Chk1-mediated G2/M checkpoint activation and may shed a new light on molecular mechanisms underlying the therapy resistance of p53-mutated tumor cells.

## RESULTS

### A dominant negative mutant of p53, p53-DD, enhances Chk1-mediated G2/M checkpoint activation induced by chemotherapeutics in hematopoietic cells

**Figure 1:**
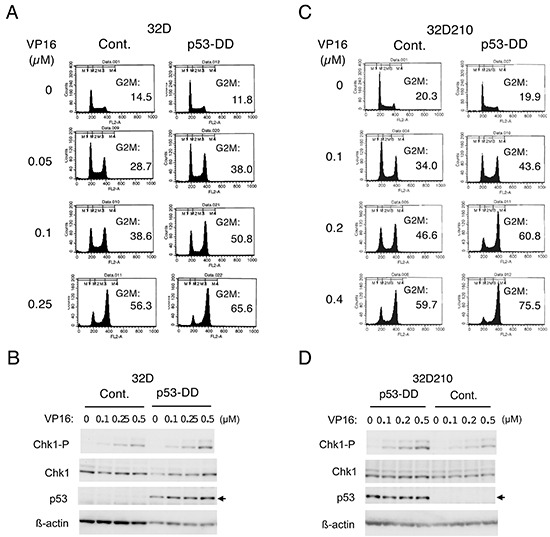
A dominant negative mutant of p53, p53-DD, enhances Chk1-mediated G2/M checkpoint activation induced by chemotherapeutics in hematopoietic cells **A.** Ton.32D/pRevTRE-p53-DD cells cultured without doxycycline (Cont.) or with 1 μg/ml doxycycline to induce expression of p53-DD (p53-DD) were treated with indicated concentrations of etoposide (VP16) for 19 h. Cells were then analyzed for the cellular DNA content by flow cytometry. Percentages of cells with the G2/M DNA content are indicated. **B.** Ton.32D/pRevTRE-p53-DD cells cultured without doxycycline (Cont.) or with doxycycline (p53-DD) were treated with indicted concentrations of etoposide (VP16) for 6 h. Cells were then lysed and subjected to Western blot analysis with antibodies against indicated proteins. The position of endogenous p53 is indicated by an arrow. Chk1-P, phospho-S345-Chk1. **C.** Ton.32D210 (Cont.) or Ton.32D210-p53-DD (p53-DD) cells were treated with indicated concentrations of etoposide (VP16) for 16 h and analyzed for the cellular DNA content by flow cytometry. Percentages of cells with the G2/M DNA content are indicated. **D.** Ton.32D210 or Ton.32D210-p53-DD cells were treated with indicated concentrations of etoposide (VP16) for 6 h and subjected to Western blot analysis with antibodies against indicated proteins.

To investigate the possible effects of p53 on Chk1-mediated G2/M checkpoint activation in hematopoietic cells, we examined Ton.32D/pRevTRE-p53-DD cells, which inducibly express a dominant negative mutant of p53, p53-DD [19, 20], as well as endogenous p53 when cultured with doxycycline (Supplementary Figure S1A). The induction of endogenous p53 is consistent with previous reports [19, 20] and supports the idea that p53-DD dominant negatively inhibits the negative feed back mechanism of p53 expression mainly involving Mdm2 [4, 5]. As shown in Figure 1A, etoposide induced accumulation of these 32D cells in the G2/M phase in a dose-dependent manner, which was enhanced by expression of p53-DD. Western blot analysis with the activation-specific Chk1 antibody further revealed that p53-DD enhanced etoposide-induced Chk1 activation in these cells (Figure 1B). It was also shown that p53 was expressed at high levels when p53-DD was inducibly expressed, while induction of endogenous p53 by etoposide was barely detectable by Western blot analysis under these conditions. Next, to examine the effects of p53 on Chk1 activation in hematopoietic cells expressing oncogenic tyrosine kinases, we utilized Ton.32D210 cells, which are 32D cells transformed by BCR/ABL and grow autonomously without IL-3 [21], and Ton.32D210-p53-DD cells expressing p53-DD (Supplementary Figure S1B). As compared with parental Ton.32D210 cells, etoposide-induced G2/M arrest and Chk1 activation were enhanced in Ton.32D210-p53-DD, as shown in Figure 1C and 1D. Essentially the same results were obtained by treatment of these cells with doxorubicin (data not shown). These observations suggest that p53 may negatively regulate Chk1-mediated G2/M checkpoint activation induced by chemotherapeutics in hematopoietic cells and their transformants.

### p53 may play a role in down regulation of Chk1-mediated G2/M checkpoint activation by inhibition of Jak2 in etoposide-treated cells

**Figure 2:**
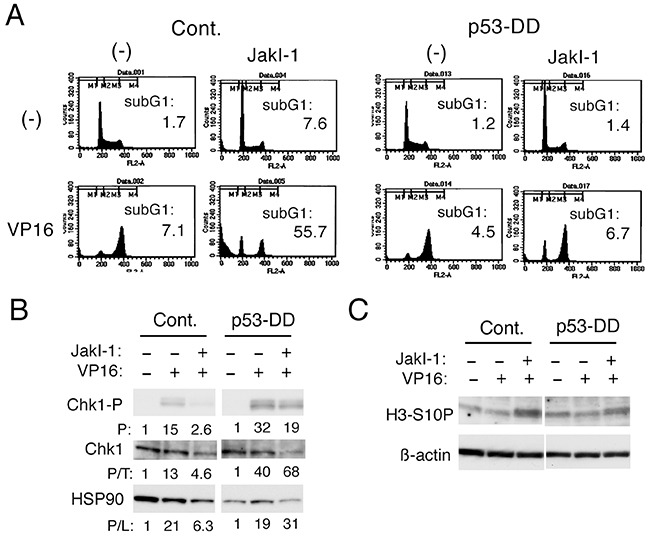
p53 may play a role in inhibition of Chk1-mediated G2/M checkpoint activation by the Jak kinase inhibitor JakI-1 in etoposide-treated cells **A.** Ton.32D/pRevTRE-p53-DD cells cultured without doxycycline (Cont.) or with 1 μg/ml doxycycline (p53-DD) were treated with or without 0.5 μM etoposide (VP16) or 0.5 μM JakI-1, as indicated, for 16 h and analyzed for the cellular DNA content by flow cytometry. Percentages of cells with the sub-G1 DNA content are indicated. **B, C.** Ton.32D/pRevTRE-p53-DD cells cultured without doxycycline (Cont.) or with doxycycline (p53-DD) were treated with or without 1 μM etoposide (VP16) or 1 μM JakI-1, as indicated, for 8 h and subjected to Western blot analysis. Abbreviations: Chk1-P, phospho-S345-Chk1; H3-S10P, phospho-S10-histone H3. Relative levels of Chk1 phosphorylated on S345 (P) and those normalized by expression levels of total Chk1 protein (P/T) as well as HSP90 used as protein loading control (P/L) were determined by densitometric analysis and are shown below each panel.

We have previously shown that inhibition of cytokine-induced activation of Jak2 signaling pathway induces apoptosis synergistically with chemotherapeutics in hematopoietic cells by down regulating Chk1-mediated G2/M checkpoint activation through mechanisms involving activation of GSK3 [16, 17]. To test the possible involvement of p53 in these processes, we examined Ton.32D/pRevTRE-p53-DD cells. In accordance with our previous reports [16, 17], inhibition of Jak2 by JakI-1 prevented etoposide-induced accumulation of these cells in the G2/M phase and drastically enhanced apoptosis, as shown in Figure 2A. Very similar results were obtained by using the Jak1/Jak2 inhibitor ruxolitinib clinically in use for myeloproliferative neoplasms (Supplementary Figure S2) [15]. In contrast, when p53-DD was induced by treatment with doxycycline in these cells, JakI-1 failed to prevent the G2/M phase arrest distinctively and to enhance apoptosis induced by etoposide (Figure 2A). Western blot analysis further showed that p53-DD, at least partly, prevented down regulation of etoposide-induced Chk1 activation by JakI-1 in these cells (Figure 2B). In accordance with our previous report [17], JakI-1 increased the expression level of H3-S10P, a mitotic marker, in Ton.32D/pRevTRE-p53-DD cells treated with etoposide (Figure 2C). As expected, p53-DD attenuated the increase in H3-S10P level induced by JakI-1 in etoposide-treated cells, thus suggesting that it protected the G2/M phase arrest under these conditions (Figure 2C). These observations suggest that p53 may play a role in down regulation of Chk1-mediated G2/M checkpoint activation by JakI-1 to enhance apoptosis in etoposide-treated hematopoietic cells.

### p53 may play a role in inhibition of Chk1-mediated G2/M checkpoint activation by imatinib or the PI3K/Akt inhibitors in BCR/ABL-expressing cells treated with chemotherapeutics

**Figure 3:**
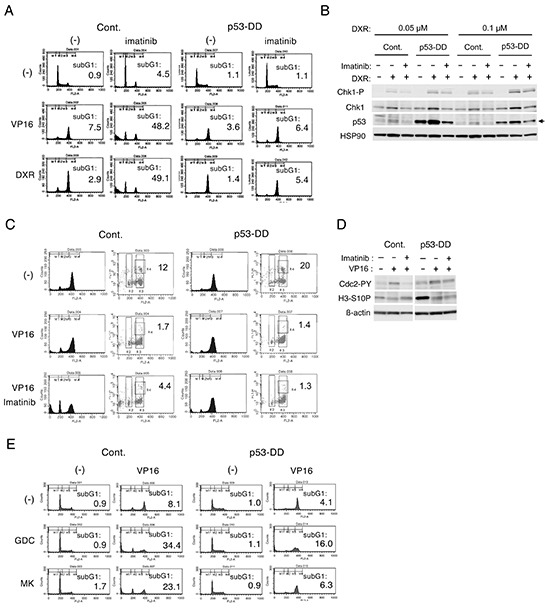
p53 may play a role in inhibition of Chk1-mediated G2/M checkpoint activation by imatinib in BCR/ABL-expressing cells treated with chemotherapeutics **A.** Ton.32D210 (Cont.) or Ton.32D210-p53-DD (p53-DD) cells were treated with or without 0.5 μM etoposide (VP16), 50 nM doxorubicin (DXR), or 1 μM imatinib, as indicated, for 24 h and analyzed for the cellular DNA content by flow cytometry. Percentages of cells with the sub-G1 DNA content are indicated. **B.** Ton.32D210 (Cont.) or Ton.32D210-p53-DD (p53-DD) cells were treated with or without indicated concentrations of doxorubicin or 3 μM imatinib, as indicated, for 8 h and subjected to Western blot analysis. The position of endogenous p53 is indicated by an arrow. **C.** Ton.32D210 (Cont.) or Ton.32D210-p53-DD (p53-DD) cells were treated with or without 1 μM etoposide (VP16) or 1 μM imatinib, as indicated, for 16 h in the presence of 50 ng/nocodazole. Cells were analyzed for the DNA content and histone H3 phosphorylated on S10 (H3-S10-P) by flow cytometry. Percentages of cells in G2/M that are positive for H3-S10-P are indicated. **D.** Ton.32D210 (Cont.) or Ton.32D210-p53-DD (p53-DD) cells were treated as described for C and subjected for Western blot analysis. Abbreviations: Chk1-P, phospho-S345-Chk1; H3-S10P, phospho-S10-histone H3; Cdc2-PY, phospho-Y15-Cdc2. **E.** Ton.32D210 (Cont.) or Ton.32D210-p53-DD (p53-DD) cells were treated with or without 0.5 μM etoposide (VP16), 3 μM GDC-0941 (GDC), or 5 μM MK-2206 (MK), as indicated, for 24 h and analyzed for the cellular DNA content by flow cytometry. Percentages of cells with the sub-G1 DNA content are indicated.

We found previously that inhibition of the aberrant tyrosine kinases involved in pathogenesis of various hematological malignancies, such as BCR/ABL, Jak2-V617F, and Flt3-ITD, also down regulated the Chk1 activation to induce apoptosis synergistically with chemotherapeutics in cells expressing these aberrant kinases [17]. To explore the significance of p53 in synergistic effects of chemotherapeutics and TKIs against these kinases, we examined the effects of p53-DD in BCR/ABL-transformed 32D cells treated with imatinib and etoposide or doxorubicin. In accordance with our previous report [17], imatinib prevented accumulation of Ton.32D210 cells treated with etoposide or doxorubicin in the G2/M phase and drastically induced apoptosis in these cells (Figure 3A). In contrast, imatinib in combination with etoposide or doxorubicin neither prevented the G2/M cell cycle arrest nor induced apoptosis unequivocally in Ton.32D210-p53-DD cells expressing p53-DD. As demonstrated in Figure 3B, Western blot analysis confirmed that p53-DD augmented Chk1 activation induced by doxorubicin similarly with that induced by etoposide demonstrated in Figure 2B. It was further revealed that imatinib down regulated doxorubicin-induced Chk1 activation more distinctively in control cells than in p53-DD-expressing cells.

Flow cytometric analysis confirmed that, while Ton.32D210-p53-DD cells as well as Ton.32D210 cells were trapped in the M phase by treatment with nocodazole alone, co-treatment with etoposide prevented the entry into M phases, as demonstrated by remarkable decreases in H3-S10P-positive cells (Figure 3C). It was further revealed that imatinib allowed the entry of etoposide-treated Ton.32D210 cells, but not Ton.32D210-p53-DD cells, into the M phase, as demonstrated by the increase in number of cells positively staining with H3-S10P. It was further confirmed by Western blot analysis that imatinib increased the expression level of H3-S10P in etoposide treated cells only in the absence of p53-DD (Figure 3D). Furthermore, imatinib inhibited the inhibitory phosphorylation of Cdc2 induced by etoposide only in the absence of p53-DD. These data indicate that inactivation of p53 may enhance the Chk1-mediated G2/M checkpoint activation involving negative regulation of Cdc2 in Ton.32D210 cells treated with chemotherapeutics to counteract synergistic induction of apoptosis by imatinib.

We previously found that inhibition of the PI3K/Akt pathway activated downstream of the aberrant tyrosine kinases also synergistically enhances the effects of chemotherapeutics in a similar manner with TKIs [17]. Thus, we next compared effects of the PI3K inhibitor GDC-0941 or the Akt inhibitor MK-2206 on BCR/ABL-driven cells with or without p53-DD expression. In accordance with our previous report [17], GDC-0941 as well as MK-2206 significantly reduced the G2/M arrest and drastically induced apoptosis in Ton.32D210 cells (Figure 3E). However, these effects were remarkably attenuated in Ton.32D210-p53-DD cells. Thus, p53 should also play an important role in synergistic induction of apoptosis by inhibition of the PI3K/Akt pathway in BCR/ABL-driven cells.

### The p53 activator nutlin-3 down regulates Chk1-mediated G2/M checkpoint activation induced by etoposide in BCR/ABL-expressing cells through different mechanisms from imatinib

**Figure 4:**
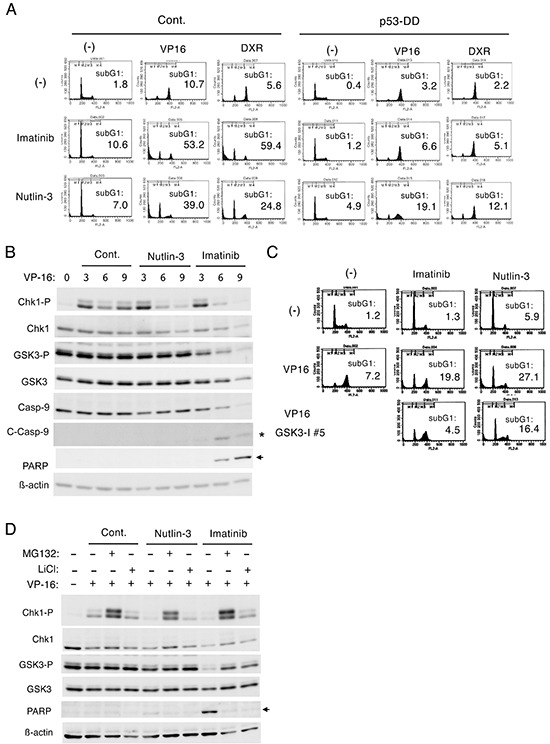
Nutlin-3 down regulates Chk1-mediated G2/M checkpoint activation induced by etoposide in BCR/ABL-expressing cells through different mechanisms from imatinib **A.** Ton.32D210 (Cont.) or Ton.32D210-p53-DD (p53-DD) cells were treated with or without 0.5 μM etoposide (VP16), 50 nM doxorubicin (DXR), 1 μM imatinib, or 5 μM nutlin-3, as indicated, for 24 h and analyzed for the cellular DNA content by flow cytometry. Percentages of cells with the sub-G1 DNA content are indicated. **B.** Ton.B210 cells expressing BCR/ABL in the presence of doxycycline were pretreated with 3 μM nutlin-3 or 5 μM imatinib, as indicated, for 1 h or left untreated as control (Cont.). Cells were then treated with 1 μM etoposide (VP16) for indicated times and subjected to Western blot analysis. The position of cleaved Caspase-9 or PARP is indicated by an asterisk or arrow, respectively. Abbreviations: Chk1-P, phospho-S345-Chk1; GSK3-P, phospho-S21/9-GSK3α/β; Casp-9, Caspase-9; C-Casp-9, cleaved Caspase-9. **C.** Ton.32D210 cells were treated with or without 1 μM etoposide (VP16), 1 μM imatinib, 5 μM nutlin-3 (5 μM), or 1 μM GSK3-I #5, as indicated, for 16 h and analyzed for the cellular DNA content by flow cytometry. Percentages of cells with the sub-G1 DNA content are indicated. **D.** Ton.B210 cells cultured with doxycycline were pretreated as described for B. Cells were then treated with 5 μM etoposide (VP16), 10 μM MG132, or 50 mM LiCl, as indicated, for 6 h and subjected to Western blot analysis.

To explore further the possibility that p53 may play a role in down regulation of Chk1-mediated G2/M checkpoint activation, we examined the effects of nutlin-3, which enhances p53 expression by inhibiting its interaction with Mdm2 [6, 7]. Similarly with imatinib, nutlin-3 inhibited the G2/M arrest and remarkably enhanced apoptosis in Ton.32D210 cells treated with etoposide or doxorubicin (Figure 4A). As expected, these effects of nutlin-3 as well as imatinib were much less significant in Ton.32D210-p53-DD, thus indicating that enhanced activation of p53 by nutlin-3 down regulated the G2/M checkpoint activation by these chemotherapeutics. In accordance with this, Western blot analysis showed that nutlin-3 as well as imatinib down regulated Chk1 activation in etoposide-treated Ton.32D210 cells (Figure 4B). It was also found that imatinib, but not nutlin-3, induced activation-specific cleavage of caspase-9 as well as caspase-mediated cleavage of PARP in etoposide-treated cells, thus suggesting that caspase-mediated cleavage of Chk1 could be involved in down regulation of Chk1 activation by imatinib, but not by nutlin-3. As expected, imatinib down regulated the inhibitory phosphorylation of GSK3β on S9 in etoposide-treated cells, which, however, was not affected by nutlin-3. Furthermore, in accordance with our previous report [17], inhibition of the G2/M arrest and enhancement of apoptosis in etoposide-treated cells by imatinib was mostly prevented by inhibition of GSK3, which, however, did not prevent the effects of nutlin-3 on these cells (Figure 4C). Consistent with this, Western blot analysis showed that, although down regulation of etoposide-induced Chk1 activation by imatinib was prevented by inhibition of GSK3 or proteasome, that by nutlin-3 was prevented only by inhibition of the latter (Figure 4D). These results suggest that, while imatinib down regulated etoposide-induced G2/M checkpoint activation mediated by Chk1 through mechanisms involving GSK3 activation and proteasomal degradation as well as activation of caspases, as we reported previously [17], the p53 activator nutlin-3 down regulated Chk1 activation through mechanisms involving proteasome degradation but not activation of GSK3 or caspases.

### Nutlin-3 down regulates Chk1-mediated G2/M checkpoint activation to induce apoptosis synergistically with etoposide in cells transformed by the T315I mutant of BCR/ABL

**Figure 5:**
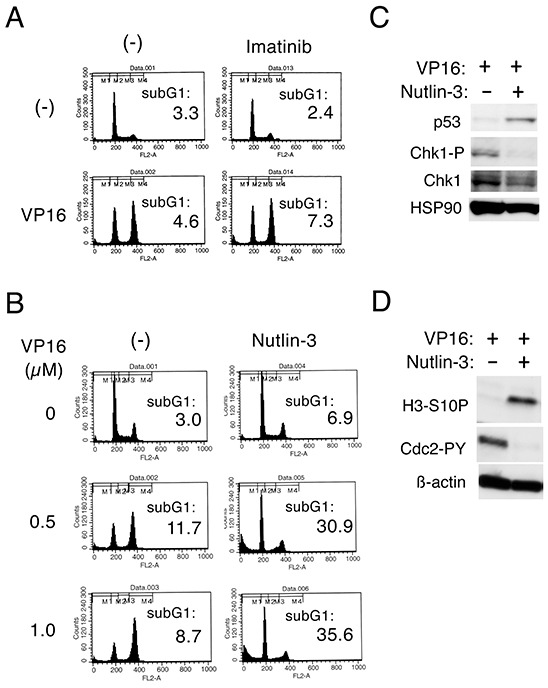
Nutlin-3 down regulates Chk1-mediated G2/M checkpoint activation to induce apoptosis synergistically with etoposide in cells transformed by the T315I mutant of BCR/ABL **A.** Ton.B210/T315I cells cultured with doxycycline in the absence of IL-3 to induce expression of the T315I mutant of BCR/ABL were treated with or without 0.5 μM etoposide (VP16) or 5 μM imatinib for 16 h and analyzed for the cellular DNA content by flow cytometry. Percentages of cells with the sub-G1 DNA content are indicated. **B.** Ton.B210/T315I cells cultured with doxycycline were treated with indicated concentrations of etoposide (VP16) with or without 5 μM nutlin-3 for 16 h and analyzed. **C, D.** Ton.B210/T315I cells cultured with doxycycline were treated with 0.5 μM etoposide for 8 h in the presence or absence of 10 μM nutlin-3, as indicated, and subjected to Western blot analysis with antibodies against indicated proteins. Abbreviations: Chk1-P, phospho-S345-Chk1; H3-S10P, phospho-S10-histone H3; Cdc2-PY, phospho-Y15-Cdc2.

To explore the clinical significance of enhancement of p53 activation in treatment of TKI-resistant leukemias, we examined the effects of nutlin-3 in cells expressing BCR/ABL with the T315I mutation, which confers resistance not only to the first generation TKI imatinib but also to the second-generation TKIs nilotinib and dasatinib [13, 14]. As expected, imatinib failed to prevent the etoposide-induced G2/M arrest to induce apoptosis in Ton.B210/T315I cell (Figure 5A), in accordance with our previous report [17]. On the other hand, nutlin-3 prevented the G2/M arrest and drastically induced apoptosis in Ton.B210/T315I cells treated with etoposide, while nutlin-3 alone did not significantly induce apoptosis in these cells (Figure 5B). It was confirmed by Western blot analyses that nutlin-3, by enhancing p53 expression and down regulating Chk1 activation, down regulated the inhibitory phosphorylation of Cdc2 and induced the mitotic marker H3-S10P expression in etoposide-treated Ton.B210/T315I cells (Figure 5C, 5D).

### p53 may down regulate Chk1-mediated G2/M checkpoint activation through its transcription-dependent function

**Figure 6:**
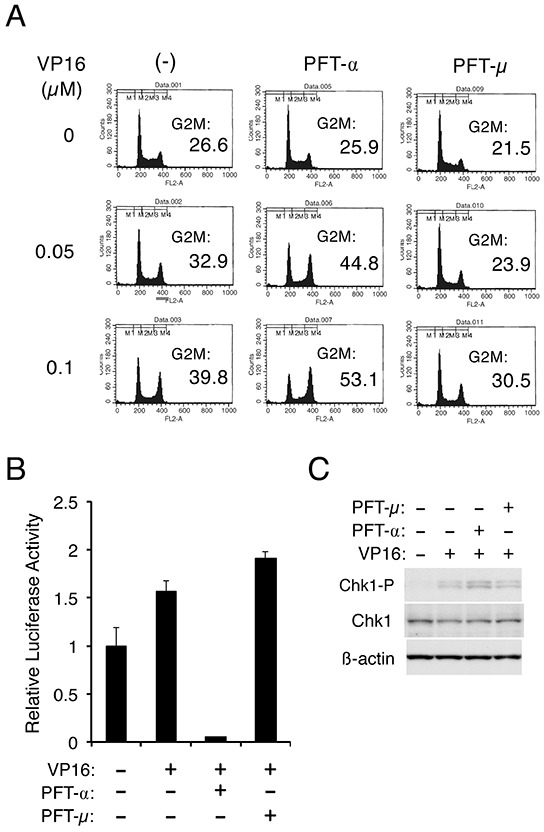
p53 may down regulate Chk1-mediated G2/M checkpoint activation through its transcription-dependent function **A.** Ton.32D210 cells were pretreated for 30 min with either 10 μM PFT-α or 5 μM PFT-μ or left untreated, as indicated. Cells were subsequently treated with indicated concentrations of etoposide (VP16) for 16 h and analyzed for the cellular DNA content by flow cytometry. Percentages of cells with the G2/M DNA content are indicated. **B.** 32Dcl3 cells were transfected with 10 μg of PG13-luc along with 0.01 μg of pRL-SV40 and cultured for one day. Cells were then pretreated with 10 μM PFT-α or 5 μM PFT-μ, as indicated, for 30 min and left untreated or treated with 0.5 μM etoposide (VP16) for 8 h, as indicated. Cells were then harvested for the dual luciferase assay. The luciferase activity was normalized by the Renilla luciferase activity and expressed as fold increase from the untreated control. The data represent averages ± SD of an experiment performed in triplicate and are representative of three repeated experiments. **C.** 32Dcl3 cells were pretreated for 30 min with either 10 μM PFT-α or 5 μM PFT-μ, as indicated, and subsequently treated for 9 h with 0.5 μM etoposide (VP16) or left untreated, as indicated. Cells were lysed and subjected to Western blot analysis with antibodies against indicated proteins. Abbreviation: Chk1-P, phospho-S345-Chk1.

To gain more insights into the mechanisms by which p53 may down regulate Chk1 activation in cells treated with chemotherapeutics, we examined the effects of pifithrin-α (PFT-α), and pifithrin-μ (PFT-μ), which inhibit transcription-dependent and -independent functions of p53, respectively [22, 23]. As shown in Figure 6A, PFT-α enhanced the G2/M arrest induced by etoposide in Ton.32D210 cells. On the other hand, PFT-μ rather down regulated the G2/M arrest in repeated experiments (Figure 6A and data not shown). The inhibitory effect of PFT-α, but not PFT-μ, on transcription-dependent activity of p53 was confirmed by luciferase reporter assays using a reporter plasmid with p53 binding sites (Figure 6B). In accordance with the enhancing effect of PFT-α on G2/M arrest, Western blot analysis revealed that PFT-α, but not PFT-μ, enhanced Chk1 activation moderately in etoposide-treated cells (Figure 6C). These data suggest that p53 may down regulate Chk1 activation through mechanisms involving its transcription-dependent function.

## DISCUSSION

The present study has shown that the dominant negative p53 mutant p53-DD or the p53 activator nutlin-3 enhanced or inhibited, respectively, the Chk1 activation critical for the G2/M arrest and survival of hematopoietic cells treated with chemotherapeutics (Figure 1, 4). These data indicate that p53 inhibits Chk1-mediated checkpoint activation in these cells. Furthermore, studies using the inhibitors for transcription-dependent and independent functions of p53, PFT-α and PFT-μ, respectively, suggested that the transcriptional regulation by p53 may be required for inhibition of the Chk1 activation (Figure 6). Previous studies have shown that Chk1 is transcriptionally down regulated by p53 directly or indirectly through induction of one of the major target gene products of p53, p21 [24–27]. Furthermore, Cummings, M. et al. [28] previously reported that transient expression of p53 in p53-negative K562 cells expressing BCR/ABL decreased the expression level of Chk1 and G2/M arrest in response to etoposide, although neither the underlying mechanism for inhibition of Chk1 expression by p53 nor the possible effect of p53 on Chk1 activation was addressed. In the present study, p53-DD or nutlin-3 affected the level of phosphorylation of Chk1 much more remarkably than its expression level. Thus, the negative effect of p53 on Chk1-mediated checkpoint activation should be caused not mainly through decrease in Chk1 expression.

It has been well established that Chk1 activation is negatively regulated mainly through its dephosphorylation by phosphatases [2]. In this regard, it should be noted that p53-inducible type-2C phosphatase Wip1 (PPM1D9) has been shown to play a role in counteracting the DNA-damage response at least partly by interacting and dephosphorylating Chk1 [2, 29]. Thus, it is possible that Wip1 or other phosphatases may be involved in negative regulation of Chk1 activation by p53. On the other hand, proteasomal degradation of phosphorylated Chk1 also plays an important role in negative regulation and termination of its activation [2]. Previous studies have revealed that ubiquitination of phosphorylated Chk1 required for proteasomal degradation is mediated mainly by two distinct E3 ligase complexes, DDB1/CUL4 and FBX6/CUL1, and is negatively regulated by the deubiquitinase USP1. The present study has implicated involvement of the proteasome pathway in inhibition of Chk1 activation by p53, because its inhibition by nutlin-3 was prevented by the proteasome inhibitor MG132 (Figure 4D). Thus, p53 may inhibit Chk1 activation by regulating expression of its target gene products involved directly or indirectly in regulation of the ubiquitin proteasome pathway as well as in dephosphorylation of Chk1. These possibilities need to be addressed in future studies to elucidate the precise molecular mechanisms involved in inhibition of Chk1 activation by p53.

We previously found that TKIs for aberrant tyrosine kinases, such as BCR/ABL and FLT3-ITD, or inhibitors for the downstream PI3K/Akt pathway synergistically enhance effects of chemotherapeutics on leukemic cells expressing these kinases by down regulating Chk1 activation [17]. This would at least partly constitute the mechanistic basis for the efficacy and validity of therapeutic strategies currently employed against these leukemias, such as Ph-positive ALL and FLT3-ITD-positive AML [13, 30, 31]. For cells expressing BCR/ABL mutants resistant to TKIs, we have revealed that the synergistic effects with chemotherapeutics could be obtained by using the p53 activator nutlin-3 or the PI3K/Akt inhibitors instead of TKIs in the present or previous study, respectively (Figure 5) [17]. Although the synergistic effect of nutlin-3 with chemotherapeutics has been established in previous studies [7], the present study has revealed that down regulation of Chk1 activation may play a role in its synergistic effect.

Importantly, the present study has also revealed that the synergistic effects of TKIs or the PI3K/Akt inhibitors with chemotherapeutics are much less remarkable in p53-deficient cells than in p53-proficient cells (Figure 3 and 4). The abnormality of p53 is not as frequently observed in hematological malignancies as in solid tumors [8]. However, it is associated with advanced stages, such as the acute phase of CML, and leads to therapy resistance with dismal prognosis in various hematological malignancies. Although TKIs could still synergistically enhance the effects of chemotherapeutics in these cells, as we previously showed in K562 cells [17], activators of mutated p53 or specific Chk1 inhibitors would more efficiently inhibit Chk1 activation to synergistically enhance the effects of chemotherapeutics in these resistant diseases. In this regard it is notable that Chk1 inhibitors have been reported to enhance the effects of DNA damaging agents more drastically in p53-deficient cells than in p53-proficient cells, although conflicting results have also been reported [32–35]. Various activators for mutated p53 [8, 36] as well as various Chk1 inhibitors [35] are currently under development for clinical use. Thus, future studies are warranted to evaluate their effects on Chk1-mediated checkpoint activation mechanisms as well as efficacies in induction of apoptosis in combination with chemotherapeutics in p53-mutated cells.

## MATERIALS AND METHODS

### Cells and reagents

Ton.B210, a clone of murine IL-3-dependent BaF3 cells transfected with a BCR/ABL cDNA under the control of a tetracycline-inducible promoter, was kindly provided by Dr. G. Daley [37]. Ton.B210 cells were cultured in 10% fetal calf serum (FCS)-containing RPMI 1640 medium supplemented either with 5 U/ml murine IL-3 or with 1 μg/ml doxycycline, which induces the expression of BCR/ABL. Ton.B210/T315I cells, which inducibly express BCR/ABL harboring the T315I mutation, murine IL-3-dependent 32Dcl3 cells, Ton.32D cells, and Ton.32D210 cells expressing BCR/ABL were described previously [21, 38, 39].

Imatinib was kindly provided by Novartis (Basel, Switzerland). Recombinant murine IL-3 was purchased from Peprotech. Doxycycline, propidium iodide (PI), PFT-α, and PFT-μ were purchased from Sigma (St Louis, MO, USA). Etoposide, doxorubicin, and LiCl were purchased from Wako (Tokyo, Japan). The Jak inhibitor JakI-1, MG132, and nocodazole were purchased from Calbiochem (La Jolla, CA, USA). Nutlin-3 and ruxolitinib were purchased from Cayman Chemicals (Ann Arbor, MI, USA) and LC Laboratories (Boston, MA), respectively. The PI3K inhibitor GDC-0941 and the Akt inhibitor MK-2206 were purchased from Chemdea (Ridgewood, NJ) and Selleck (Houston, TX), respectively. GSK3-inhibitor #5 (GSK3 I-#5) [40] was synthesized and kindly provided by Dr. H. Kagechika. DiOC6 was purchased from Invitrogen (Carlsbad, CA, USA).

Antibodies against Chk1 (SC8408), p53 (SC6243), and HSP90 (SC13119) were purchased from Santa Cruz Biotechnology (Santa Cruz, CA). Antibodies against phospho-S345-Chk1 (CS2348), phospho-S21/9-GSK3α/β (CS9331), GSK3β (CS9315), cleaved Caspase-3 (CS-9661), Caspase-9 (CS-9508), cleaved Caspase-9 (CS-9509), and phospho-Y15-Cdc2 (CS9111) were purchased from Cell Signaling Technology (Beverly, MA). Antibodies against phospho-S10-histone H3 (06-570) and PARP (SA-250) were purchased from Millipore (Billerica, MA) and Biomol (Plymouth Meeting, PA), respectively. Anti-β-actin was purchased from Sigma (St. Louis, MO).

### Expression plasmids, transfection, and infection

A retrovirus expression plasmid, pRevTRE-p53-DD, was described previously [18]. An expression plasmid, pTRE-Tight-p53-DD, was constructed by subcloning the *Bam*HI/*Sal*I fragment coding for p53-DD from pBABE-hygro-p53-DD [20], a gift from Bob Weinberg (Addgene plasmid #9058) into pTRE-Tight (Clontech; Mountain View, CA).

Transfection of the retroviral vector pRevTRE-p53-DD into PLAT-A cells and infection of Ton.32D or Ton.B210 cells was performed as described previously [39]. After selection with hygromycin, infected cells were used as Ton.32D/TRE-p53-DD or Ton.B210/TRE-p53-DD cells. Transfection of pTRE-Tight-p53-DD into Ton.32D210 cells by electroporation with pMAM2-BSD (Funakoshi, Tokyo, Japan), followed by selection and limiting dilution, was performed as described previously [39]. Selected clones were found to express p53-DD with or without doxycycline by Western blot analysis. A selected clone, Ton.32D210-p53-DD, expressing a high level of p53-DD, was used for subsequent experiments.

### Flow cytometric analyses for cell cycle and apoptosis

For flow cytometric analyses of cell cycle and apoptosis, cells were incubated with Krishan's reagent (0.05mg/ml PI, 0.1% Na citrate, 0.02mg/ml ribonuclease A, 0.3% NP-40) and analyzed as described previously [16]. For multivariate flow cytometric cell cycle analysis, cells were simultaneously stained for DNA content with PI and for histone H3 phosphorylated on S10 using specific primary antibodies and FITC-conjugated goat F(ab')2 fragment anti-rabbit IgG (H+L) antibody (IM0833) from Beckman Coulter (Miami, FL, USA), essentially as described previously [17].

### Immunoprecipitation and immunoblotting

Cells were lysed and subjected to immunoprecipitation and immunoblotting, essentially as described previously [41]. The results shown are representative of experiments repeated at least three times.

### Luciferase reporter assays

A luciferase reporter plasmid with p53 binding sites, PG13-luc [42] was a gift from Bert Vogelstein (Addgene plasmid #16442). A control Renilla luciferase plasmid, pRL-SV40, was purchased from Promega (Madison, WI, USA).

Luciferase reporter assays of transiently transfected cells were performed essentially as described previously [18, 43]. In brief, cells were electroporated with PG13-luc as well as pRL-SV40 and treated with or without the p53 inhibitors and etoposide as described before harvesting for the luciferase assay using Dual-Luciferase Reporter Assay System (Promega) according to the manufacturer's instructions.

## SUPPLEMENTARY FIGURES


