# Supplementary material for: Down regulation of Chk1 by p53 plays a role in synergistic induction of apoptosis by chemotherapeutics and inhibitors for Jak2 or BCR/ABL in hematopoietic cells
Source: Oncotarget. 2016 Jun 6;7(28):44448–61. doi: 10.18632/oncotarget.9844 (PMC5190110; doi:10.18632/oncotarget.9844)
Supplement: Supplementary file 1 [file oncotarget-07-44448-s001.pdf]

SUPPLEMENTARY FIGURES

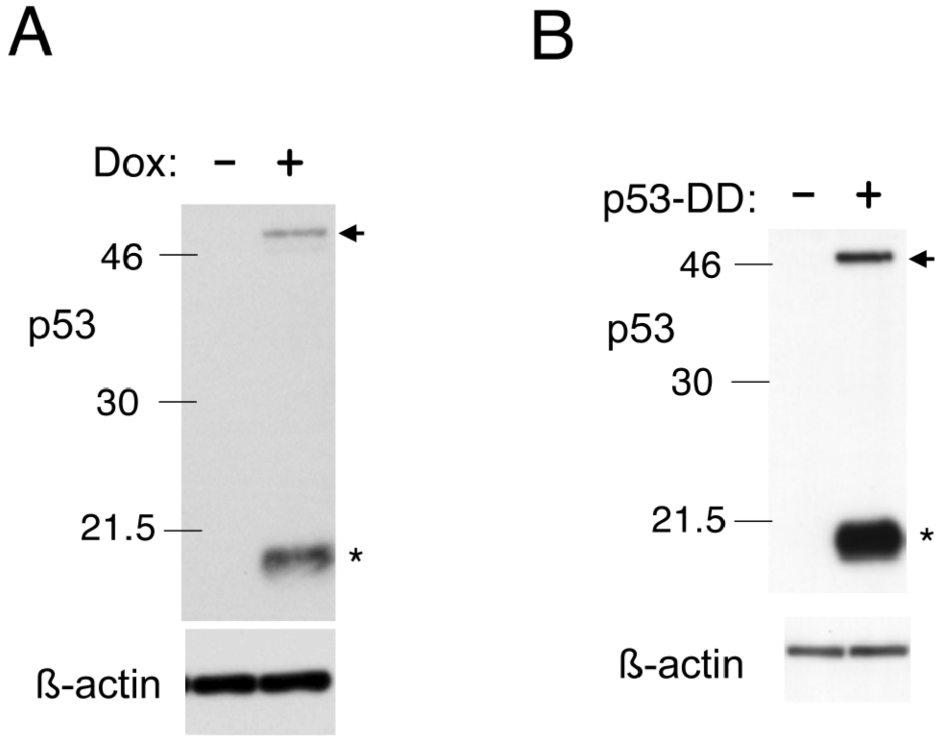

Supplementary Figure S1: Expression of the dominant negative mutant p53-DD in Ton.32D/pRevTre-p53-DD and Ton.32D210-p53-DD cells. A. Ton.32D/TRE-p53-DD cells cultured with or without 1 µg/ml doxycycline (-) or with 1 µg/ml doxycycline (+) to induce expression of p53-DD, as indicated, were lysed and subjected to Western blot analysis with antibodies against indicated proteins. The position of endogenous p53 or p53-DD is indicated by an arrow or asterisk. **B.** Ton.32D210 cells (-) and Ton.32D210-p53-DD cells (+) were subjected to Western blot analysis.

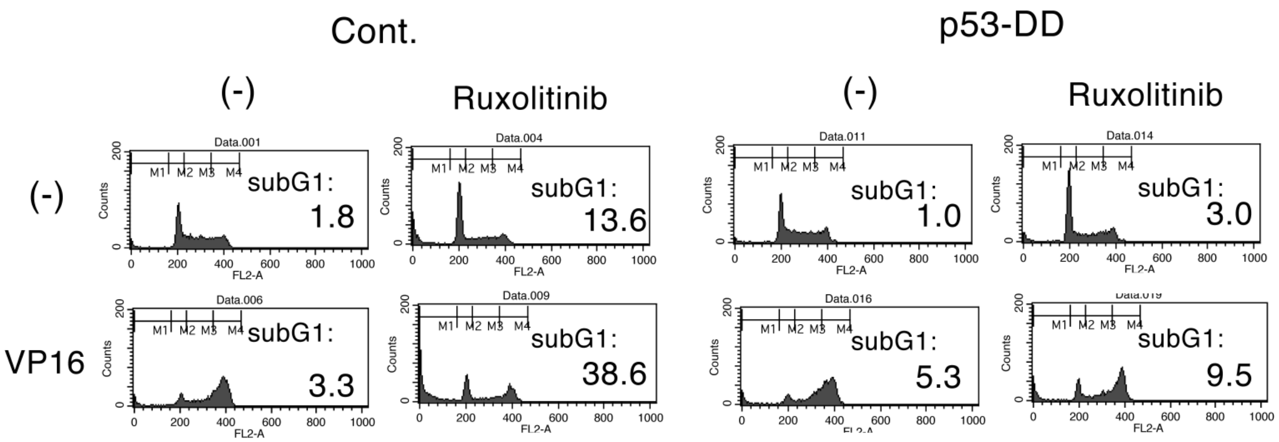

Supplementary Figure S2: p53 may play a role in inhibition of Chk1-mediated G2/M checkpoint activation by ruxolitinib in etoposide-treated cells. Ton.32D/pRevTRE-p53-DD cells cultured without doxycycline (Cont.) or with 1 µg/ml doxycycline (p53-DD) were treated with or without 0.5 µM etoposide (VP16) or 1 µM ruxolitinib, as indicated, for 16 h and analyzed for the cellular DNA content by flow cytometry. Percentages of cells with the sub-G1 DNA content are indicated.
